# Complicated diverticular disease of the colon, do we need to change the classical approach, a retrospective study of 110 patients in southeast England

**DOI:** 10.1186/1749-7922-3-5

**Published:** 2008-01-24

**Authors:** Abdulzahra Hussain, Hind Mahmood, Gokulakkrishna Subhas, Shamsi EL-Hasani

**Affiliations:** 1 Department of general surgery, Princess Royal University Hospital, Kent, UK; 2 General surgery department, Farnborough common, Orrington, BR6 8ND, Kent, UK

## Abstract

**Background:**

Complicated diverticular disease of the colon imposes a serious risk to patient's life, challenge to surgeons and has cost implications for health authority. The aim of this study is to evaluate the management outcome of complicated colonic diverticular disease in a district hospital and to explore the current strategies of treatment.

**Methods:**

This is a retrospective study of all patients who were admitted to the surgical ward between May 2002 and November 2006 with a diagnosis of complicated diverticular disease. A proforma of patients' details, admission date, ITU admission, management outcomes and the follow up were recorded from the patients case notes and analyzed. The mean follow-up was 34 months (range 6–60 months)

**Results:**

The mean age of patients was 72.7 years (range 39–87 years). Thirty-one men (28.18 %) and Seventy-nine women (71.81%) were included in this study. Male: female ratio was 1:2.5.

Sixty-eight percent of patients had one or more co-morbidities. Forty-one patients (37.27%) had two or more episodes of diverticulitis while 41.8% of them had no history of diverticular disease.

Eighty-six percent of patients presented with acute abdominal pain while bleeding per rectum was the main presentation in 14%. Constipation and erratic bowel habit were the commonest chronic symptoms in patients with history of diverticular disease. Generalized tenderness was reported in 64.28% while 35.71% have left iliac fossa tenderness. Leukocytosis was reported in 58 patients (52.72%).

The mean time from the admission until the start of operative intervention was 20.57 hours (range 4–96 hours). Perforation was confirmed in 59.52%. Mortality was 10.90%. Another 4 (3.63%) died during follow up for other reasons.

**Conclusion:**

Complicated diverticular disease carries significant morbidity and mortality. These influenced by patient-related factors. Because of high mortality and morbidities, we suggest the need to target a specific group of patients for prophylactic resection.

## Background

Complicated diverticular disease is defined as diverticulitis with associated abscess, phlegmon, fistula, stricture or obstruction, bleeding, or perforation [1-3]. Hinchey's classification of acute diverticulitis is including a phlegmon (stage Ia), localized abscesses (stages Ib and II), free perforation with purulent (stage III) or feculent peritonitis (stage IV) [4]. Management of the diverticular disease of the colon has seen progressive success owing to the advances of the diagnostic methods, intensive care settings, minimal access techniques and surgical experience. While there is little debate about the best treatment for mild episodes and/or very severe episodes, uncertainty persists about the optimal management for intermediate stages (Ib and II) [4].

Many studies reported variable rates of mortality and morbidities for patients presented with complicated diverticulitis. The morbidity could reach 44% [3] while the mortality rates range from as low as1% to16.7% [5,6]. More serious colonic perforation with generalized fecal peritonitis is associated with a mortality of over 50% in most series [7]. Mortality is found to be accurately evaluated by Colorectal-Physiologic and Operative Severity Score for the enUmeration of Mortality and morbidity (POSSUM) [8].

Because of the high rates of morbidities and mortality, some authors advise prophylactic resection of the affected colonic segment depending on the outcomes and follow-up of people who had recurrent episodes of diverticulitis [9-12]

The aim of this study is to evaluate the outcomes of management of complicated diverticular disease of the colon in a district NHS hospital and to explore the current strategies of its treatment.

## Methods

This is a retrospective study of 110 patients who have been admitted to the surgical ward of Princess royal university hospital/Kent between May 2002 and November 2006 with diagnosis of complicated diverticular disease. The spectrum of clinical picture was ranged from stage 1–4 according to Hinchey's classification.

A proforma of patients' details, mode of presentation, clinical findings, radiographic and laboratory findings, preoperative co-morbidities, type of complicated diverticulitis, operative intervention, morbidity, mortality, length of stay, operative or conservative management outcomes, intensive care admission and the follow up were recorded from the patients' case notes.

The exclusion criteria included patients who diagnosed with other bowel pathologies such as cancer, inflammatory bowel disease and mechanical obstruction due to other causes.

### The criteria for diagnosis and classification

The diagnosis of the stage of complicated diverticulitis (Hinchey's classification) depended on the history, examination, laboratory findings, imaging (ultrasound &CT scan) and colonoscopy.

The outcomes were specified using statistical analysis. The statistical significance was considered at (*P *< 0.05).

## Results

### Patients' demography

The mean age of the patients was 72.76 years (range 39–87 years). Thirty-one men (28.18 %) and seventy-nine women (71.81%). Male: female ratio was 1:2.5.

Forty-one (37.27%) patients had one or more episodes of diverticulitis.

### History of diverticular disease

About 20.90% of patients had one episode of diverticular disease. Forty-one patients (37.27%) had two or more episodes of diverticulitis while 41.8% of patients had no history of diverticular disease.

### Co-morbidity

Sixty-seven patients (60.90%) had one or more co-morbidities. Cardiovascular disease is the commonest co-morbidity (62 patients-56.36 %). Eight diabetic patients (7.27%), 28 (25.45%) patients with connective tissue and vascular disease and twelve patients (10.90%) had different co-morbidities.

### Clinical findings

**Figure 1:**
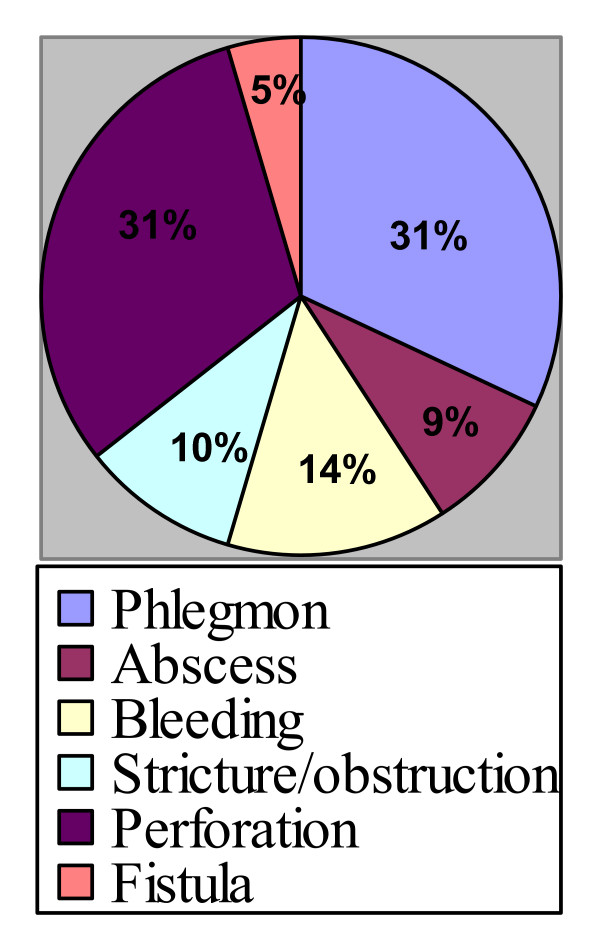
Presentation of complicated diverticular disease.

Phlegmon was the commonest presentation (31.81%, no = 35) followed by perforation (30.90%, no = 34), bleeding, stricture and fistula (See figure 1)

Eighty-six percent of patients were presented with acute abdominal pain while rectal bleeding was the main symptom in14%. Constipation and erratic bowel habit were the commonest chronic symptoms. Generalized tenderness was reported in 72 patients (65.45%) while left iliac fossa tenderness was reported in 38 patients (34.54 %). Leukocytosis was reported in 58 patients (52.72%). Sigmoid colon was the commonest site for complicated diverticultis in 91%. Other sites in order of frequency were descending colon, transverse colon, ascending colon and the ceacum.

### Surgical treatment

The mean waiting time from the admission until the start of operative intervention was 20.57 hours (range 4–96 hours). Operative intervention was reported in 57 (51.81%) patients. Hartman's procedure was performed in 32 (56.14%), sigmoid colectomy and ileostomy in 15(26.30%) and proximal colostomy in 5 (8.77%) patients.

Segmental resection and division of the fistula was performed for 5 (8.77%) patients while percutaneous drainage was performed in 10(9.09%) patients with pericolic abscesses. Seven (6.36%) patients who were presented primarily with phlegmon and bleeding underwent laparotomy because they were progressed to a worse stage during hospitalization while one of them developed perforation following colonoscopy.

### Conservative treatment

Fifty-three (48.18%) Patients were treated by intravenous fluids, analgesia, blood and antibiotics and showed an excellent response.

### Intensive care unit ICU

Four (7.01%) patients were admitted to ICU before operation while 25(43.85%) patients were admitted postoperatively. The mean ICU length of stay was 7.36 days (range 1–28 days)

### Histopathology

Perforated diverticular disease was confirmed microscopically in 15(44.11%) out of 34 patients underwent emergency surgery for perforated diverticular disease. The length of the resected colon ranged from 8–93 cm with average of 17.59 cm. Diverticular disease was confirmed in all resected specimens.

### Morbidity

Fifty-four postoperative complications were reported with overall morbidity of 49.09%. Respiratory complications (22.22%) was the commonest morbidity followed by cardiac (16.66%) and wound complications (14.81%).(See table 1).

**Table 1:** Morbidity

Complications	No	%
Ileus	3	05.55
Wound complications	8	14.81
Burst abdomen	1	01.85
Necrosis of the stoma	1	01.85
Pelvic abscess	1	01.85
Septicemia	3	05.55
Fistula	2	03.70
Incisional hernia	4	07.40
Renal	5	09.25
cardiac	9	16.66
Respiratory	12	22.22

Total	54	100

### Mortality

Overall mortality of this cohort was 14.53%. Twelve (10.90%) patients with perforation died (one intra-operative death, 8 post-operative deaths and 3 deaths for non-operative treatment). Another 4 (3.63%) patients died during follow up because of other reasons.

### History of diverticular disease

**Figure 2:**
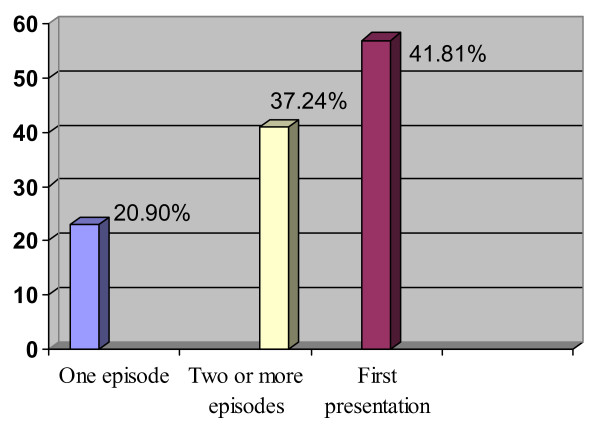
History of diverticular disease.

Twenty-three (20.90%) patients had a history of one episode of diverticular disease while forty-one (37.27%) patients had two or more episodes. Fifty-seven (41.81%) patients reported with complicated diverticulitis as their first presentation. (See figure 2)

### Hospital stay

The mean hospital stay was 16.40 days (range 2–61 days). Long stay was associated with perforated diverticulitis and postoperative morbidity.(*p *< 0.05)

## Discussion

Diverticular disease of the colon is a common problem in developed countries with its prevalence increasing with age, varying from < 10 % in those < 40 years of age, to an estimated 50–66% of patients > 80 years of age [13]. The major factor in the development of diverticulosis is lack of adequate fiber intake [14,15]. A quarter of patients with diverticulitis will develop potentially life-threatening complications including bleeding, perforation, fistulae, obstruction or stricture [16].

Fifty-seven patients of our series were subjected to emergency surgical treatment (See table 2). This included patients with perforation, peritonitis, fistula, obstruction and strictures. Hartman's operation was performed in about 56% of patients who required surgery. This is consistent with other studies, which suggest Hartman's as a safe and effective procedure especially in emergency [17-19]. Patients who presented with bleeding and phlegmon were treated conservatively with intravenous fluids, blood, and optimization of coagulation profile and showed good primary response. However, seven (6.36%) of these patients who were presented primarily with phlegmon and bleeding underwent laparotomy because they were progressed to a worse stage during hospitalization while one of them developed perforation following colonoscopy.

**Table 2:** Type of operation

Type of operation	No	%
Hartman's procedure	32	56.13
Sigmoid colectomy and ileostomy	15	26.30
Diversion only	05	08.77
Segmental resection and division of the fistula	05	08.77

Total	57	100

The overall complication rate following surgical and conservative management in our series was 49.09% (54 complications). This was less than other series, which reported a higher morbidity rate [20]. Non-surgical respiratory and cardiac complications were the commonest morbidity while wound complications were the commonest surgical morbidity (See table 1). The preoperative co morbidity of our series was 60.90 %. The presence of such co morbidities, especially older age, collagen-vascular disease, pulmonary disease, and cardiovascular disease, were all found to significantly increase the risk of development of post surgical complications[21].

The association between high morbidity and perforated diverticultis was confirmed in our study however, no significant association between morbidity and other forms of complicated diverticulitis was found.

Only 21% of perforated diverticulitis had a previous history of diverticular disease while 58.2% of other less severe forms such as phlegmon, bleeding and localized pericolic abscess had such positive history. This statistically significant association (*P *value = 0.00) was also confirmed in other studies [22].

The rate of progression of diverticular disease to complicated diverticulitis is varied. After one episode of diverticulitis, one-third of patients have recurrent symptoms; after a second episode, a further third have a subsequent episode while perforation is commonest during the first episode of acute diverticulitis [23]. An acute complicated presentation of the disease occurs in a minority of patients range from 15–25% [24-26].

Variable mortality rate was reported in literature and ranged from 4–16% while high mortality figure of 50% was confirmed in cases of perforation with generalized fecal peritonitis [27-31]. Our perioperative mortality rate was 10.90%. The majority of deaths associated with perforated diverticulitis, which was reported in 66.66% while pericolic abscess was confirmed in 33.33% of deaths (See table 3). Four patients (3.63%) who were diagnosed with perforated diverticulitis were very high-risk patients because of age and co morbidity and a decision was made to treat them conservatively. Three (2.72%) of them died while one patient responded to this approach. Another four (3.63%) patients died because of other pathologies making the overall mortality figure of 14.53%. The mean age of deaths was 79.33 years and majority were women (91.16%).

**Table 3:** Risk factors for perioperative mortality

	Patients											
Variable	1	2	3	4	5	6	7	8	9	10	11	12

Cardiovascular			*	*	*		*	*	*	*	*	*
Respiratory		*				*					*	
Connective tissue disorder								*			*	
Diabetes									*			
Immune suppression	*							*				
Renal					*	*				*		
Perforation	*	*	*	*	*		*			*	*	
Abscess						*		*	*			*
Bleeding												
Fistula												
Obstruction/stricture												
Phlegmon												
Age (year)	86	80	60	80	83	85	86	65	84	82	77	84
Sex:F = Female, M = Male	F	F	F	F	F	F	F	F	F	M	F	F

Surgery for acute complications of diverticular disease of the sigmoid colon carries significant rates of morbidity and mortality, the latter of which occurs predominantly in cases of severe co morbidity. Therefore, postoperative mortality and morbidity are largely driven by patient-related factors [32,33].

There are no clear guidelines for the indication of elective surgery in sigmoid diverticular disease although in patients with recurrent diverticulitis or with fistulae, the long-term results of surgery are satisfactory [34]. Moreover, precise criteria, which indicate elective or prophylactic surgery in patients with diverticular disease of the colon, are anatomical deformity of the sigmoid, including stenosis and fistulae, recurrent acute diverticulitis, prior diverticulitis with perforation and recurrent gastrointestinal bleeding. Many authors accepted these criteria [35-39].

Some authors suggest laparoscopic lavage and drainage of perforated acute diverticulitis as a promising alternative to more radical procedures [40]. Furthermore, minimal access surgery as elective treatment of sigmoid diverticular disease may be associated with reduced postoperative morbidity and hospital stay. It is increasingly replacing open resection as standard surgery for recurrent and complicated diverticulitis at certain centers [41-44].

Identification of patients who are at risk to develop complicated diverticulitis especially perforation, is very important to reduce mortality and morbidity. In our study cardiovascular, connective tissue disorders and diabetes were the commonest co morbidities associated with complicated diverticulitis. The factors that associated with mortality following complicated diverticulitis in our series were age, sex (female patient), cardiovascular, respiratory problems and immune suppression. Perforation and pericolic abscess were also risk factors for mortality.

These parameters and their effects, which have been confirmed in literatures, should be taken in consideration during management of complicated diverticular disease [45-47].

The risk factors for perioperative mortality in our series included the age (about 75% of patients were above 80 year), female patients (91.6% were female), cardiovascular disease (75%), Renal and respiratory disease (25%), Immune suppression and diabetes (16% and 08% respectively) (see table 3). Therefore, to reduce future mortality we suggest that prophylactic segmental colonic resection should be advised especially for female patients with recurrent episodes of diverticulitis, who are less than 70 year old, who have associated cardiovascular, respiratory or renal co morbidities. Severity of episode, without doubt is another inclusion criterion. This is generally consistent with the principle that prophylactic segmental colonic resection must be based on a balance assessment of risk factors including age, severity of attacks and their recurrence [48,49].

As acute surgical emergency, acute complicated diverticulitis resembles acute appendicitis to a large extent except for certain features. The similarity included also the high morbidity and mortality associated with these two conditions. Although major advances in the management of complicated diverticulitis have been well documented and currently widely practiced, however the overall management plan included prophylactic resection, is still under continuous evolution and less radically treated in comparison to the acute appendicitis. We think that acute diverticulitis and its complications should be treated effectively, as acute appendicitis is treated. Therefore, all patients with acute complicated diverticulitis should be followed-up and certain group of these patients has to be advised for further surgical intervention. This will need more clarification and definition of the indication criteria for prophylactic resection. According to our findings, these criteria should include the age limit, sex (females), and associated co-morbidities.

Our study has defined important risk factors for mortality, and therefore we are advising a more radical approach for management of this disease. However, further larger study is suggested to confirm our conclusions.

## Conclusion

Complicated diverticulitis carries a significant mortality and morbidity. Identification of high-risk patients to develop complications is important to reduce mortality and morbidity. Preoperative co-morbidities are the major influencing factor for peri-operative mortality and morbidity. Emergency Hartman's operation is the procedure of choice to treat majority of perforated diverticulitis. Prophylactic segmental colonic resection may be suggested for selected group of patients.

## Competing interests

The author(s) declare that they have no competing interests.

## Authors' contributions

A Hussain wrote the article, participated in the sequence alignment and drafted the manuscript. G Subhas carried out the data collection. H Mahmood participated in the sequence alignment, the design of the study and performed the statistical analysis. S El-hsani conceived of the study, and participated in its design and coordination and helped to draft the manuscript. All authors read and approved the final manuscript.
